# Distinct MDCT imaging features to differential diagnosis of hepatic paragonimiasis and small hepatocellular carcinoma

**DOI:** 10.18632/oncotarget.16197

**Published:** 2017-03-15

**Authors:** Sheng Zhang, Si-Ming Xie, Yong-Hua Chen, Xu-Bao Liu, Gang Mai

**Affiliations:** ^1^ Department of Pancreatic Surgery, West China Hospital of Sichuan University, Chengdu 610041, Sichuan Province, China; ^2^ Department of General Surgery, The First Affiliated Hospital of Xinxiang Medical College, Weihui 453100, Henan Province, China; ^3^ Department of Gastrointestinal Surgery, West China Hospital of Sichuan University, Chengdu 610041, Sichuan Province, China; ^4^ Department of Hepatobiliary Surgery, The People's Hospital of Deyang, Deyang 618000, Sichuan Province, China

**Keywords:** paragonimiasis, hepatocellular, hepatic, MDCT, carcinoma

## Abstract

We used multi-row detector computed tomography (MDCT) to identify the distinguishing characteristics of hepatic paragonimiasis and small hepatocellular carcinoma lesions. We analyzed a cohort of 60 patients, of which 26 had hepatic paragonimiasis and 34 with a small (≤ 3cm) hepatocellular carcinoma. MDCT detected 65 lesions that were retrospectively reviewed and analyzed based on their imaging features. Both groups showed distinct MDCT imaging features that could contribute to an accurate diagnosis. In the paragonimiasis group, 75% (21/28) lesions were located in the hepatic subcapsular region, whereas only 10.8% (4/37) of lesions in the hepatocellular carcinoma group were subcapsular. Most hepatic paragonimiasis lesions (57.1%; 16/28) also showed characteristic tubular or tunnel features that were not present in hepatocellular carcinomas. Further, 71.4% (20/28) paragonimiasis lesions were rim enhanced with irregular tract-like non-enhanced internal areas with a characteristic target loop, while 94.6% (35/37) of small hepatocellular carcinoma lesions showed homogenous enhancement in the arterial and venous phase. In addition, the period CT values for hepatic paragonimiasis were less than those of hepatic carcinomas (P<0.001). These clinically significant findings illustrate the diagnostic features that enable one to distinguish hepatic paragonimiasis from small hepatocellular carcinomas.

## INTRODUCTION

Paragonimus westermani is a trematode parasite that causes pulmonary or extrapulmonary granulomatous disease in humans infected due to ingestion of raw or incompletely cooked freshwater crab or crayfish infected with metacercaria [1, 2]. Generally, paragonimiasis lesions are situated in the lungs, but ectopic infestation could occur in the brain [3], intra-peritoneal cavity [4, 5], sub-cutaneous tissue [6] and the liver [7, 8]. Although the involvement of the liver is rare, CT features of hepatic paragonimiasis has been reported in the literature. For lesions with diameter **≤**3 cm, it is difficult to distinguish between hepatic paragonimiasis and hepatocellular carcinoma (HCC). Therefore, early diagnosis is necessary to avoid surgery.

The aim of this study was to clarify the Multi-row Detector Computed Tomography (MDCT) features of hepatic paragonimiasis and small hepatocellular carcinoma lesions **≤**3cm and identify the distinguishing features.

## RESULTS

Among the 60 patients that were studied, 26 were cases of paragonimiasis and 34 were HCC cases. Regarding gender, the paragonimiasis group consisted of 16 males and 10 females whereas the HCC group had 20 males and 14 females. There was no statistical significance between the two groups (P=0.83). The average age of the paragonimiasis patients was 41.58±12.16 years, whereas, it was 45.24±10.17 years for the HCC patients (P=0.21). The comparative information on WBC, eosinophilia, total bilirubin, γ-globulin and tumor markers is presented in Table 1 and no statistical difference was observed between the two groups. The size of lesions was comparable between the two groups (2.18±0.60 *vs* 2.08±0.61cm; P=0.50) (Table 2). Comparsion of the CT values, as listed in Table 2, showed that the CT values of the paragonimiasis group were significantly lower in each phase than the HCC group (P<0.001) and could be used to distinguish hepatic paragonimiasis and small hepatocellular carcinoma.

**Table 1:** Characteristics of patients

	Hepatic paragonimiasis	Hepatocellular carcinoma	P values^*^
**Age**	41.58±12.16	45.24±10.17	0.21
**Sex**			
**Male**	16(26.7%)	20(33.3%)	
**Female**	10(16.7%)	14(23.3%)	0.83
**Temperature (°C)**	36.53±0.35	36.56±0.35	0.73
**WBC (x 10^9^)**	7.08±3.38	5.79±1.59	0.08
**Eosinophils (× 10^8^)**	0.44±0.30	0.54±0.28	0.17
**γ-Globulin (g/L)**	26.13±3.97	25.59±3.24	0.57
**Total Bilirubin (μmol/L)**	16.10±9.54	18.10±10.54	0.45
CA199 (units/ml)*	14.30±10.63	16.57±12.00	0.45
AFP (ng/ml)*	18.30±13.22	24.97±17.75	0.10

* tumors markers measured by electrochemiluminescence.

** P<0.05 statistical significance.

**Table 2:** Comparison of size and phase of the CT values

	Hepatic paragonimiasis	Hepatocellular carcinoma	P values
**Size(cm)**:	2.18±0.60	2.08±0.61	0.50
**Plain scan(Hu)**:	25.21±4.69	44.62±7.57	<0.001
**Arterial phase(Hu)**:	30.46±5.69	61.03±7.16	<0.001
**Venous phase(Hu)**:	36.54±7.34	75.86±6.66	<0.001

Data are expressed as Mean±SD; P<0.05 is considered statistically significant.

In the paragonimiasis group, 75% (21/28) of the lesions detected on MDCT were subcapsular. The lesions were distributed among segments VIII (28.6%; 8/28), VII (21.4%; 6/28), VI (17.9%; 5/28) and V (14.3%; 4/28), respectively. 57.1% (16/28) of the lesions were tubular or tunnel shaped (Figure IIA, IIB). All the lesions were hypoechoic. 71.4% (20/28) of the lesions showed rim enhancement with irregular tract-like non-enhanced internal areas and formed characteristic target loop whereas 17.9% (5/28) lesions were homogenously enhanced and 11% (3/28) lesions were non-enhanced.

**Figure 1:**
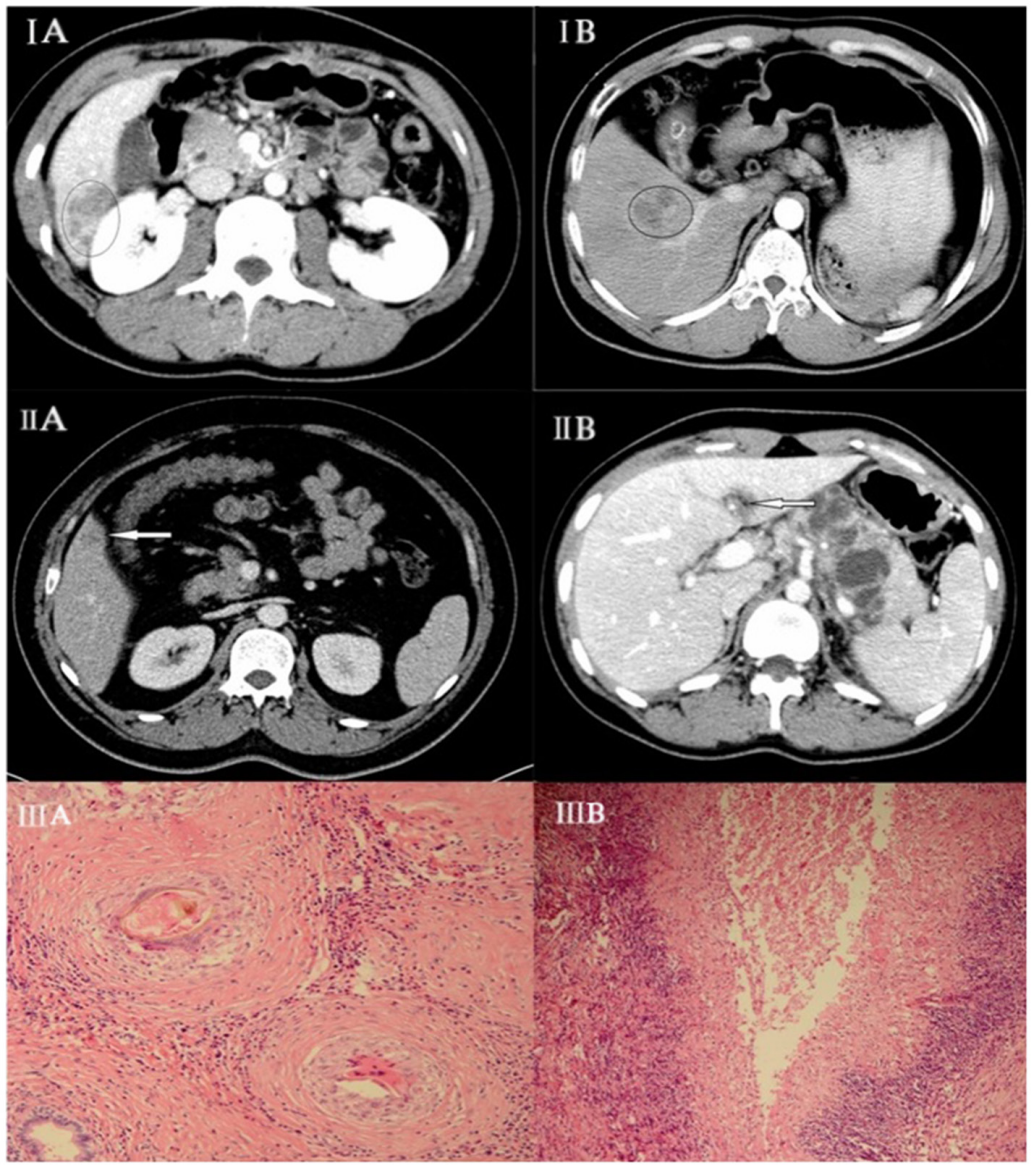
(IA) Hepatic paragonimiasis in a 50-year-old male patient Enhanced MDCT scan at portal venous phase shows heterogeneous enhancement. **(IB)** Hepatocellular carcinoma in a 45-year-old female. Enhanced MDCT scan at portal venous phase shows homogeneous enhancement. **(IIA)** Hepatic paragonimiasis in a young male. Enhanced MDCT scan at portal venous phase shows solid, ring-enhancing with forming target loop as indicated by the arrow. **(IIB)** Image shows tubular formation in early phase of paragonimiasis as indicated by the arrow. **(IIIA)** Pathological finding showing an egg being engulfed by a macrophage with coagulative necrosis within the lesion surrounded by **(IIIB)** infiltration of a large number of barrier-like arrayed epithelioid cells.

In the HCC group, 37 lesions were detected on MDCT. 59% (22/37) of HCC lesions were located in segments III, IV or VII and only 10.8% (4/37) lesions were subcapsular. All the lesions were solid and regular shaped. In the CT scan, all the lesions were hypoechoic with 94.6% (35/37) showing homogenous enhancement in arterial and venous phase (Figure IB).

## DISCUSSION

The species P. westermani was discovered in the lungs of a dead tiger by Kerbert in 1878 [9] and is endemic to Asian, African and South American countries such as China, Japan, Liberia, Nigeria and Venezuela [10]. At least 293.8 million people are at risk of infection from paragonimus parasites and that includes 195 million from China [11]. Epidemiological data shows increased paragonimiasis in some regions of China [12] with an epidemic declared in the Sichuan province [13]. Since we treated hepatic paragonimiasis patients often, we had rich experience of treatment and access to research materials.

The diagnosis for paragonimiasis in the liver is based on the pathological analysis of the biopsy that considers (1) coagulative or liquefactive necrosis within the lesion; (2) infiltration of a large number of eosinophils forming chronic eosinophilic abscesses and sporadic distribution of Charcot-Leyden crystals; and (3) hyperplasia of granulomatous and fibrous tissue around the lesion (Figure IIIA, IIIB).

Once the liver is infected, it is difficult to distinguish paragonimiasis with HCC. Although FDG PET/CT scans are currently used to diagnose hepatic parasites, FDG activity during malignancy renders them inaccurate [14, 15]. Previous studies had demonstrated that the MDCT features of hepatic paragonimiasis were favorable for its specific diagnosis [16, 17]. In this study, the right liver lobe was most commonly involved, probably due to its close proximity to the duodenum and the migration route of the worm. Besides, the hepatic subcapsular region was the most common site of paragonimiasis as reported by Lee and colleagues [18]. The lesions were initially located mainly in the subcapsular region because the parasites penetrated the intestinal wall and migrated through the peritoneal cavity to the liver after perforating the Glisson capsule. In addition, the migration route of the worm formed a tubular or tunnel sign [19, 20]. These features were rare in the hepatocellular carcinoma. Hence, MDCT was superior to FDG PET/CT in finding morphological changes that are specific to paragonimiasis.

Pathologically, the feature of these lesions was conglomerated eosinophilic abscesses in which the unenhanced area represented necrotic debris, numerous eosinophils and Charcot–Leyden crystals. On the other hand, the enhanced septa in the cystic lesions and the enhanced area in the solid lesions represented granulomatous inflammation and hyperplasia of the fibrous tissue. Our study showed that 71.4% (20/28) of the lesions were rim enhanced with irregular tract-like nonenhanced internal areas, forming the characteristics of the target loop in hepatic paragonimus. Comparatively, 94.6% (35/37) lesions of the small hepatocellular carcinoma showed homogenous enhancement in the arterial and venous phase. However, quantitative study of the enhanced mode of hepatic paragonimiasis and hepatocellular carcinoma to evaluate the differential diagnosis was not possible.

From a clinical standpoint, our study shows that the diagnosis of paragonimiasis should be established according to the clinical manifestation, laboratory test results and characteristic imaging findings. The MDCT imaging features with low CT values may accurately distinguish hepatic paragonimus from small liver carcinoma lesions and help avoid surgery in many cases.

## METHODOLOGY

A cohort of 60 patients at West China Hospital of Sichuan University from July 2009 and June 2013 were included in this study (This study was approved by the Regional Ethics Committee of our hospital and all patients signed informed consents). The post-operative pathology confirmed if lesions with a diameter **≤**3cm were hepatic paragonimiasis or hepatocellular carcinoma. We reviewed the MDCT examination results of the 60 patients and collected other information like age, gender, white blood cell counts, eosinophilia, total bilirubin, γ-globulin, tumor marker data and patient history. All the patients in this study were residents of the Sichuan province in China, which is an endemic area of paragonimiasis, especially P. skrjabini. 28.3% (17/60) patients had a history of eating crayfish, whereas, 50% (30/60) patients had a history of hepatitis B virus infection.

All patients underwent abdominal plain and enhanced MDCT examination. The scan technique varied owing to the retrospective nature of the study. All images were obtained when the patient was in supine position and during inspiration. First, plain MDCT scan was performed followed by injection of the iodinated contrast agent (Iohexol injection, 100ml: 30g, GE Healthcare Ireland) through the cubital vein using a 20-gauge needle and an automatic injector (2–3mL/kg of contrast agent at a rate of 3ml/s). Further, arterial and portal venous phase scans were initiated at 25s and 50s after the start of the injection. In general, examinations were performed using a spiral technique with 5–10mm collimation and 5–10mm reconstruction intervals. Forty-three out of the sixty patients were scanned using a 64 multi-detector CT (Brilliance 64; Philips Healthcare, Best, Netherlands) with an x-ray tube voltage of 120kV, 200mA current, 512×512 matrix size, 2×2 pixel size and 64×0.625mm collimator width. The seventeen other patients were scanned using a16 multi-detector CT (Brilliance 16; Philips Healthcare, Best, Netherlands) with 120kV voltage, 250mA current, 512 ×512 matrix size, 2×2 pixel size and 16×1.5mm collimator width.

Two readers retrospectively collected and studied the segmental location, size, shape, density and degree and pattern of enhancement and the CT values [Four regions of interest (ROIs) of 5mm^2^ were selected for every lesion and the average values were calculated]. Statistical analysis was performed by the SPSS 19.0 Inc., (Chicago, IL, USA) software. The count data was analyzed by the chi-square test and the measurement data was analyzed by the Student's t test. A P<0.05 was considered significant.
